# Combination of Docking-Based and Pharmacophore-Based Virtual Screening Identifies Novel Agonists That Target the Urotensin Receptor

**DOI:** 10.3390/molecules27248692

**Published:** 2022-12-08

**Authors:** Na Li, Lin Yin, Xi Chen, Jiamin Shang, Meidai Liang, Li Gao, Guifen Qiang, Jie Xia, Guanhua Du, Xiuying Yang

**Affiliations:** 1 State Key Laboratory of Bioactive Substance and Function of Natural Medicines and Beijing Key Laboratory of Drug Target and Screening Research, Institute of Materia Medica of Peking Union Medical College, Beijing 100050, China; 2 Shanxi Bethune Hospital, Shanxi Academy of Medical Sciences, Tongji Shanxi Hospital, Third Hospital of Shanxi Medical University, Taiyuan 030032, China; 3 National Institute of Hospital Administration, Beijing 100050, China; 4 Modern Research Center for Traditional Chinese Medicine, The Key Laboratory of Chemical Biology and Molecular Engineering of Ministry of Education, Shanxi University, Taiyuan 030032, China; 5 State Key Laboratory of Bioactive Substance and Function of Natural Medicines, Department of New Drug Research and Development, Institute of Materia Medica, Chinese Academy of Medical Sciences and Peking Union Medical College, Beijing 100050, China

**Keywords:** urotensin receptor, urotensin-II, docking-based virtual screening, pharmacophore-based virtual screening, drug screening

## Abstract

The urotensin receptor (UT receptor), a G-protein-coupled receptor mediating urotensin-II and urotensin-II-related peptide signaling in the urotensinergic system, has multiple pharmacological activities. However, there is no drug targeting the UT receptor currently in clinical use, and the discovery of new leads is still important. The complete crystal structure of the UT receptor has not yet been resolved and a screening strategy combining multiple methods can improve the accuracy and efficiency of drug screening. This study aimed to identify novel UT receptor agonists using a combination of docking-based, pharmacophore-based, and cell-based drug screening. First, the three-dimensional structures of the UT receptor were constructed through single-template, multi-template homologous modeling and threading strategies. After structure evaluation and ligand enrichment analysis, a model from the threading modeling was selected for docking-based virtual screening based on stepwise filtering, and 1368 positive compounds were obtained from our compound library. Second, the pharmacophore models were constructed using known ligands targeting the UT receptor for pharmacophore-based virtual screening. A model was selected after model validation, and 300 positive compounds were retrieved. Then, after intersecting the results of two different virtual screening methods with 570 compound entities from our primary screening, 14 compounds were obtained. Finally, three hits were obtained after in vitro confirmation. Furthermore, preliminary evaluation of the hits showed that they influenced glucose consumption. In summary, by integrating docking-based, pharmacophore-based, and in vitro drug screening, three new agonists targeting the UT receptor were identified which may serve as promising therapeutic agents for urotensinergic system disorders.

## 1. Introduction

The urotensin receptor (UT receptor), also known as G-protein-coupled receptor 14 (GPR14), was first discovered as a specific receptor of urotensin-II (U-II) and urotensin-II-related peptide (URP) by GlaxoSmithKline in 1999 [1]. The UT receptor and endogenous ligands are widely expressed in various tissues from fish to mammals [2,3]. Clinical studies have shown that U-II/UT expression levels are closely associated with a variety of diseases, such as cardiovascular [4], liver [5], and kidney diseases [6]. It has been suggested that U-II may play a key role in glucose and fat metabolism [7,8,9]. Polymorphisms in the U-II gene are associated with risk of insulin resistance and diabetes [7]. A study showed that serum triglyceride and cholesterol levels were elevated in homozygous mice with deletion of the Uts2r gene compared to wild-type controls [8]. Our previous study found that chronic U-II administration improved glucose tolerance in obese mice [9]. Thus, the activation of the urotensinergic system is of great importance for the regulation of glucose and lipid metabolism diseases. However, no agonists of the UT receptor have been approved for clinical practice. Most reports on agonists of the UT receptor are peptides, such as P5U [10] and UPG84 [11]. However, peptides have the disadvantage of being unstable, and thus a few small-molecule agonists targeting the UT receptor have been identified, but their pharmacological activities are uncertain [12,13,14]. Therefore, there is an urgent need to identify the novel indications of diseases related to UT receptor agonism and new agonists with therapeutic efficacy.

At present, computer-aided virtual drug screening has been widely used for its time and cost savings [15,16]. The virtual drug screening models include the docking model [17,18], pharmacophore model [19,20], quantitative structure–activity relationship (QSAR) model [21], etc. Currently, virtual screening is also important in targeting G-protein-coupled receptor (GPCRs) drug discovery [17,22,23]. Despite GPCRs being membrane proteins, there is a lack of high-resolution three-dimensional (3D) crystal structures of GPCRs. Until now, the 3D crystal structure of the UT receptor has not been solved, and it is particularly important to establish a homologous model and perform virtual screening. Several studies have reported using virtual screening for ligands of the UT receptor [24,25,26]. Stefanie Flohr et al. [24] identified nonpeptidic UT receptor ligands by pharmacophore-based virtual screening (VS) by a pharmacophore model derived from structure–activity relationships and nuclear magnetic resonance studies on U-II. Elodie Lescot et al. [25] established a single-template homology 3D model of the UT receptor using the structure of bovine rhodopsin and described the mode of ligand interaction within the receptor binding site for docking-based virtual screening (DBVS). Soo-Kyung Kim et al. [26] discovered UT ligands through DBVS by multi-template homology modeling and experimental validation. However, drug discovery targeting the UT receptor still has poor sensitivity and efficiency and may yield biased results [27]. Thus, we propose a strategy of combining multiple approaches to help overcome this challenge. In this study, we applied a virtual screening strategy based on the combination of receptor docking and ligand pharmacophore and combined it with in vitro cellular screening to identify novel agonists targeting the UT receptor with the aim of improving drug screening efficiency.

## 2. Results

### 2.1. Construction of a Benchmarking Dataset Targeting UT Receptor and Ligand Enrichment Analysis

**Figure 1:**
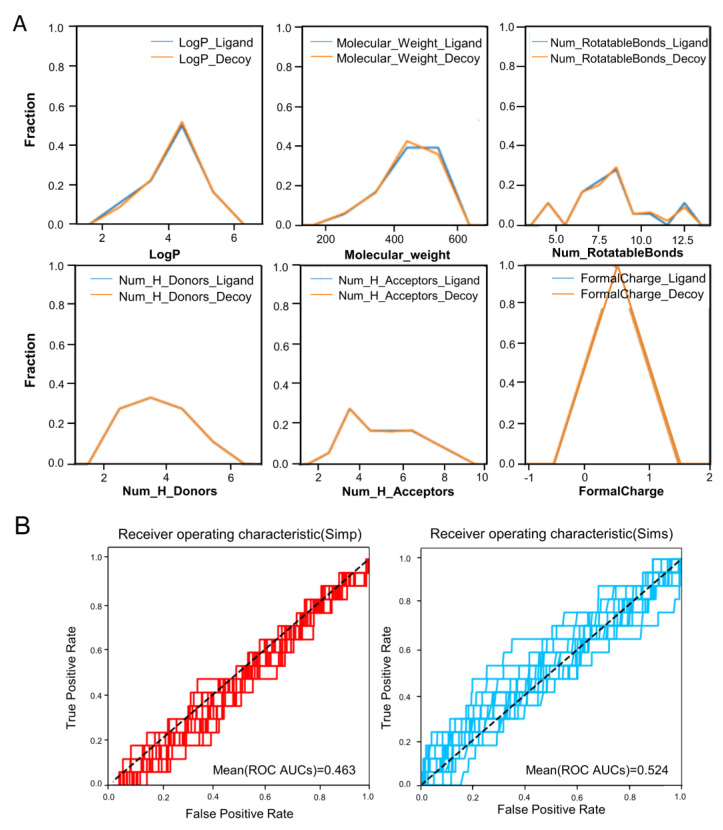
The quality evaluation of constructed MUBD-UT dataset (196 ligands targeting UT receptor and 702 randomly generated decoys). (**A**) The property distribution curves of the constructed MUBD-UT dataset for the six physicochemical properties LogP, molecular weight, formal charge, the number of rotatable bonds, H donors, and H acceptors. (**B**) The ROC curves. Left: Simp. Right: Sims.

To perform an unbiased DBVS targeting the UT receptor, we first used an online Python GUI application (MUBD-Decoy Maker 2.0) to generate a maximal unbiased benchmarking dataset. A total of 196 ligands (Table S1) collected from the ChEMBL database and others were put into the program to obtain a benchmarking dataset targeting the UT receptor, which was named MUBD-UT dataset. It included ligands targeting the UT receptor and unbiased decoys. The distribution curves of the MUBD-UT dataset matched well on each of the properties, i.e., LogP, MW, RBs, and nFC (Figure 1A). They spanned a similar range in terms of the maximum and minimum values for both ligands and decoys, and the peaks of curves were located at the same interval, especially AlogP. Meanwhile, the mean value of the ROC area under the curve (AUC) was close to the line of 0.5, indicating that it is rather challenging to distinguish ligands and decoys based on physicochemical properties (Figure 1B). This maximal-unbiased dataset was deemed to be of no “artificial enrichment” and thus ideal for the evaluation of the virtual screening model.

### 2.2. Homology Modeling of the 3D-Model of the UT Receptor

To construct a reliable homology 3D structure of the UT receptor, three strategies were evaluated, including single-template modeling, multi-template modeling, and threading modeling. First, the templates in single-template modeling were selected according to a literature review and BLAST search that were similar to the UT receptor sequence, and a total of 10 single-template models were established (Table S2). The template PDB numbers were 4N6H, 5DHG, 5DHH, 4EJ4, 5NDD, 4DJH, 4RWA, 4RWD, 2RH1, and 6G79. Second, the multi-template modeling structures were constructed by GPCR-SSFE 2.0, which is a web server to identify the key sequence and structural motifs of class A GPCRs, including guide template selection and available homology models. By querying the GPCR-SSFE 2.0 database with a given UniProt entry name (UR2R_Human), we obtained the multiple sequence alignments of the entire serpentine domain and template suggestions. In the multi-template modeling, eight templates were evaluated and chosen for each helix modeling. Their PDB numbers were 4EJ4, 4EA3, 4MBS, 5NDD, 2RH1, 4NTJ, 5UEN, and 1U19 (Figure S1, Table S3). Third, in threading modeling, an online platform (I-TASSER) that implements the algorithms for protein structure and function predictions was used for UT receptor modeling. After the target sequence was submitted, the top five models of UT receptor with the highest C-scores were selected for the next step evaluation. The C-score is the confidence score used in I-TASSER to evaluate the quality of the predicted models. The C-score is typically in the range of −5 to 2, and a higher C-score indicates higher confidence in the model. The C-scores of the top five models were −0.33, −0.54, −3.49, −1.09, and −4.51, respectively.

These modeling protein structures were evaluated by Ramachandran plot, overall quality factor, and verify 3D (Table S4). There were 11 models chosen for secondary evaluation, of which 5 were single-template models, 5 were threading models, and 1 was a multi-template model. To select the desirable models for subsequent study, the models were preliminarily docked with 196 known ligands using the LibDock module in Discovery Studio (DS) (Table S5). Finally, four models were selected for the following study in accordance with the docking results, and their RMSD comparison is shown in Table S6, including one 4N6H-based model from single-template modeling (STM-1, renamed Model 1), one multi-template model (MTM-1, renamed Model 2), and two models among five threading models (TDM-4, renamed Model 3; TDM-5, renamed Model 4).

### 2.3. Validation of Generated Homology Model

**Figure 2:**
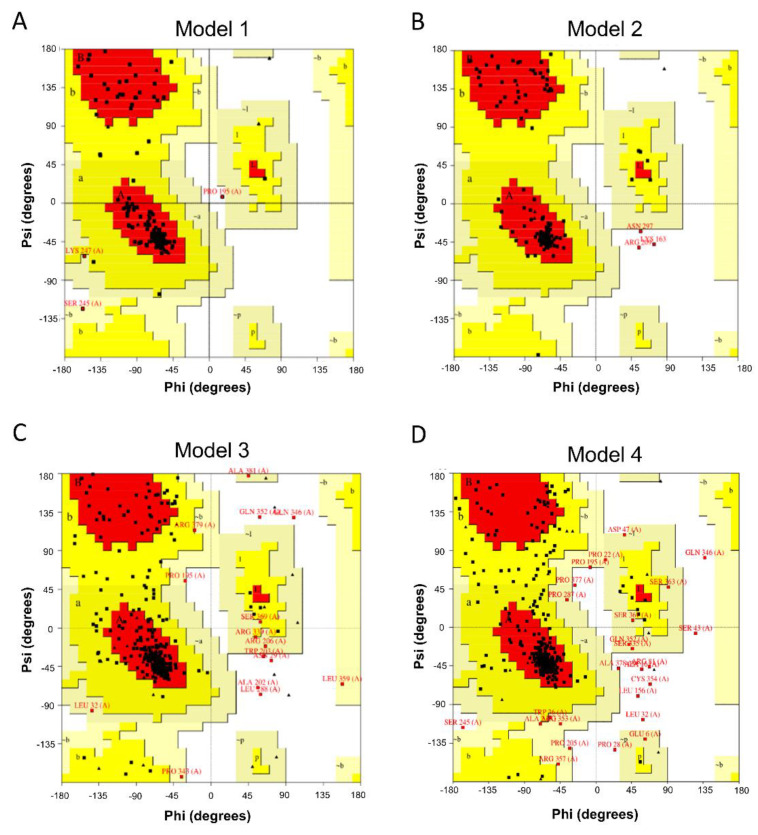
Ramachandran plots of 4 three-dimensional models constructed by three different modeling methods. (**A**) The single-template model generated with 4N6H (Model 1): 95.6% residues in most favored regions; 3.6% residues in d regions; 0.8% residues in generally allowed regions. (**B**) The multi-templates model (Model 2): 93.9% residues in most favored regions; 4.9% residues in d regions; 1.2% residues in generally allowed regions. (**C**) First threading model (Model 3): 81.4% residues in most favored regions; 14.6% residues in d regions; 2.2% residues in generally allowed regions; 1.9% residues in disallowed regions. (**D**) Second threading model (Model 4): 75.5% residues in most favored regions; 18.6% residues in d regions; 3.1% residues in generally allowed regions; 2.8% residues in disallowed regions.

Additionally, known ligands and decoys were used to evaluate these models to reduce the occurrence of false-positives. PROCHECK [28] is a program that relies on Ramachandran plots for structure verification, by which a good model needs to meet a criterion of over 95% of its residues in allowed regions, including the most favored region, additional allowed regions, and generally allowed regions. As shown in Figure 2, the results from PROCHECK showed that Model 1 had 100% of its residues in the allowed regions, and Model 2 had 98.8% of its residues in the allowed regions. Although Models 3 and 4 performed less well in the most favored regions, they were still acceptable models, with 98.1% and 97.2% of their residues in the allowed regions. Therefore, regarding the percentage distribution of the residues, the quality of these models met the evaluation requirements.

ERRAT works by analyzing the statistics of nonbonded interactions between different atom types, with quality factor of 85 or more indicating a better-quality model [29]. In this study, the overall quality factors of models 1, 2, 3, and 4 were 98.17, 95.15, 93.97, and 88.98, respectively, implying that the nonbonded interactions in the constructed models were acceptable. Collectively, the four UT receptor homology models all met the requirements for subsequent molecular docking.

**Figure 3:**
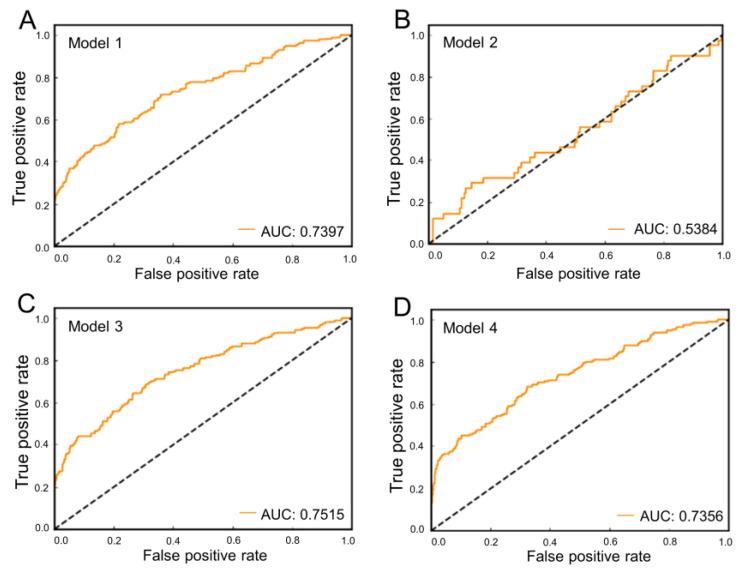
The ROC curves of four generated models to evaluate the ability of the models to distinguish active ligands and decoys. (**A**) The single-template model generated with 4N6H (Model 1). (**B**) The multi-templates model (Model 2). (**C**,**D**) Two different threading models (Model 3 and 4).

A total of 898 small molecules (196 UT receptor ligands and 702 decoys) were used for the enrichment analysis. Molecular docking of these ligands and decoys was carried out to assess the ability of the UT receptor homology modeling models to identify positive ligands among the highly random compounds. Figure 3 shows the ROC curves generated from docking between homology models and the benchmarking dataset. The values of ROC AUC indicate the ability of models to identify positive compounds and their reliability, and they were 0.7393, 0.5384, 0.7515, and 0.7356, respectively. Through analysis of the results of model validation, Model 3 had the best performance and was used in the following DBVS.

### 2.4. Pharmacophore Generation and Validation

There were 14 diverse molecules selected in the training set to generate pharmacophore models, containing active compounds with corresponding EC_50_ values less than 1 μM (Table S7). In this study, the Common-Feature-Pharmacophore module in DS 2018 was used to construct the HipHop pharmacophore model based on the common characteristic of small molecular ligands. A total of 10 pharmacophore models were generated. The rank value represented the matching degree between the molecules in the training set and the pharmacophore models. The values of the 10 generated pharmacophore models all exceeded 110 points, indicating that the molecules selected for the training set had common characteristics (Table S8).

**Figure 4:**
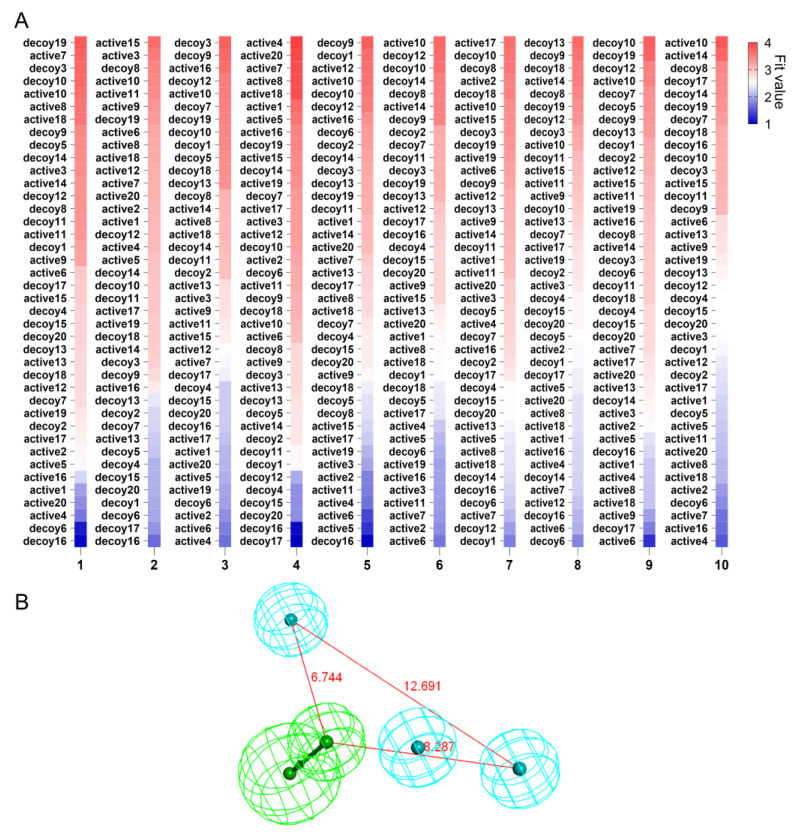
Generation of pharmacophore for urotensin receptor. (**A**) Heatmap with labels of 10 generated pharmacophore models evaluated by the test set. (**B**) The pharmacophore model used in the pharmacophore-based virtual screening. Color codes: green, hydrogen bond acceptor; light blue, hydrophobic group.

After 10 HipHop models were generated, a test set composed of 20 active and 20 inactive ligands was used to identify the optimal HipHop model. The heatmap based on the fit value could represent the ability of the models to discern active compounds, in which the warm color represents the pharmacophore with a higher degree of ligand identification, while the cool color represents the opposite. By ranking the fit value, we found that the HipHop-Hypo04 model performed best in distinguishing active compounds from the dataset, in which 9 of the top 10 compounds were active compounds, except only 1 of the bottom 10 compounds was the active compound (Figure 4A). Finally, the pharmacophore model HipHop-Hypo04, composed of three hydrophobic and one hydrogen bond acceptor was selected for subsequent pharmacophore-based VS (Figure 4B).

### 2.5. Drug Screening and Discovery of Novel Lead Compounds

**Figure 5:**
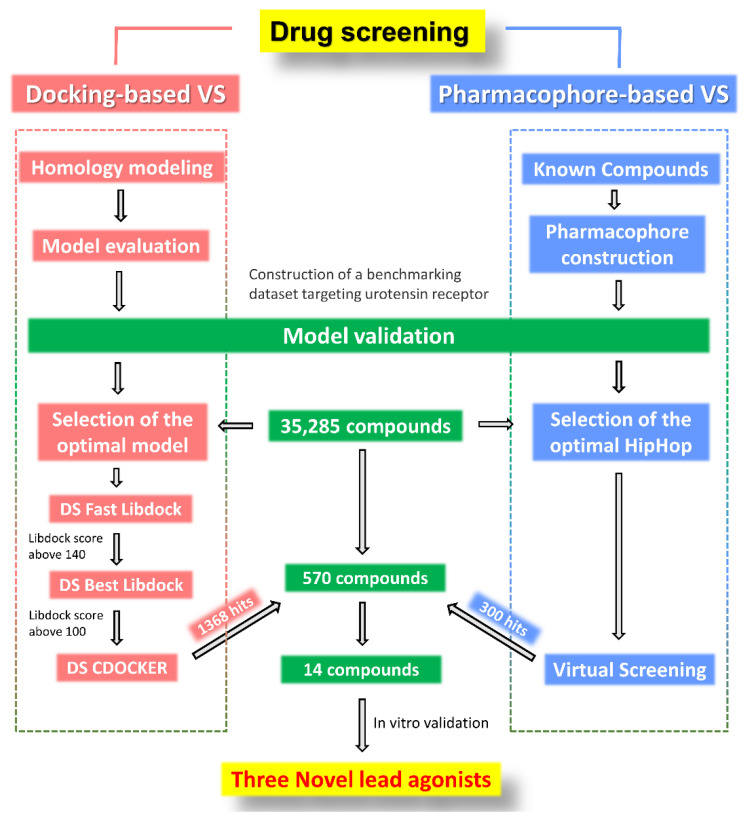
Flowchart diagram for the virtual and in vitro screening for potential lead agonists targeting urotensin receptor.

As shown in Figure 5, we used three approaches to discover novel compounds targeting UT receptor, including DBVS, pharmacophore-based VS, and cellular in vitro drug screening.

First, a total of 35,285 compounds from our in-house compound library were docked with Model 3, by which a stepwise filtering protocol was used. In the first stage, compounds were docked using Fast LibDock with default parameters where a total of 29,871 hits were obtained. From these hits, we selected 8851 compounds with LibDock scores greater than 140. These 8851 compounds were further docked using Best LibDock and smart minimizer algorithms, and 1368 hits were obtained by applying the maximum LibDock score to more than 100. Second, HipHop-Hypo04 was used as a 3D structural query to screen 35,285 compounds, and 300 hit compounds were retrieved. In addition, we selected 570 compound entities that met the requirements of preliminary screening and in-depth evaluation for cellular-based screening.

**Figure 6:**
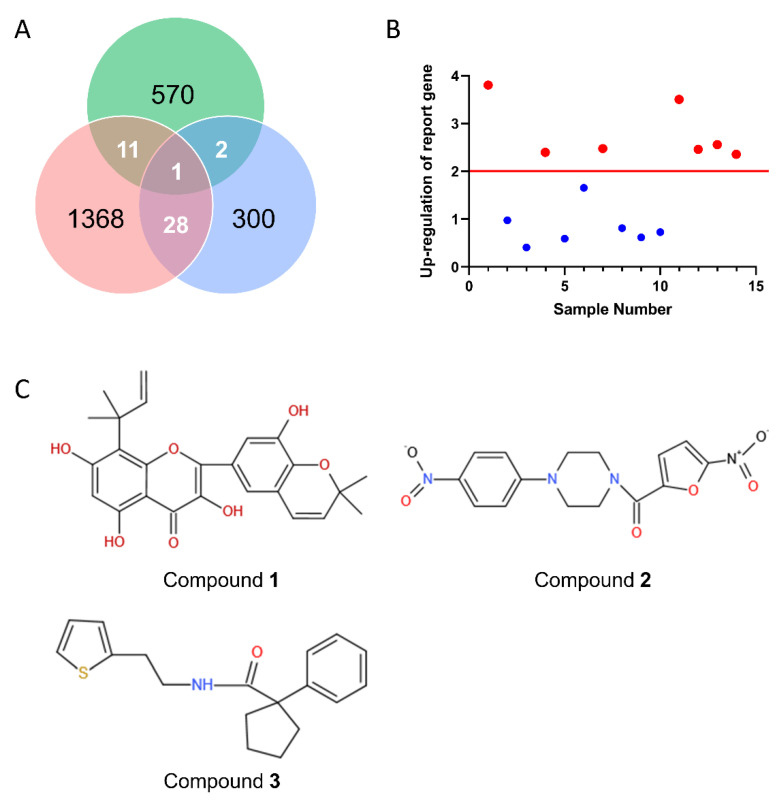
The discovery of urotensin receptor agonists. (**A**) The Venn diagram of the virtual screening results with compound entities. Color codes: green, in vitro cellular screening; light red: docking-based virtual screening; blue: pharmacophore-based virtual screening. (**B**) Preliminary screening results of cellular screening in vitro. (**C**) The chemical structures of the three hit compounds.

Intersecting the results of virtual screening with 570 compounds, there were 14 compounds that either positively docked with the generated receptor structure or shared the pharmacophoric features derived from known potential ligands targeting the UT receptor (Figure 6A). Subsequently, a secondary evaluation was conducted in vitro, and seven positive compounds whose luciferase activity was upregulated two times were obtained (Figure 6B). After further evaluation, there were three compounds with a dose–effect relationship (Figure 6C).

**Figure 7:**
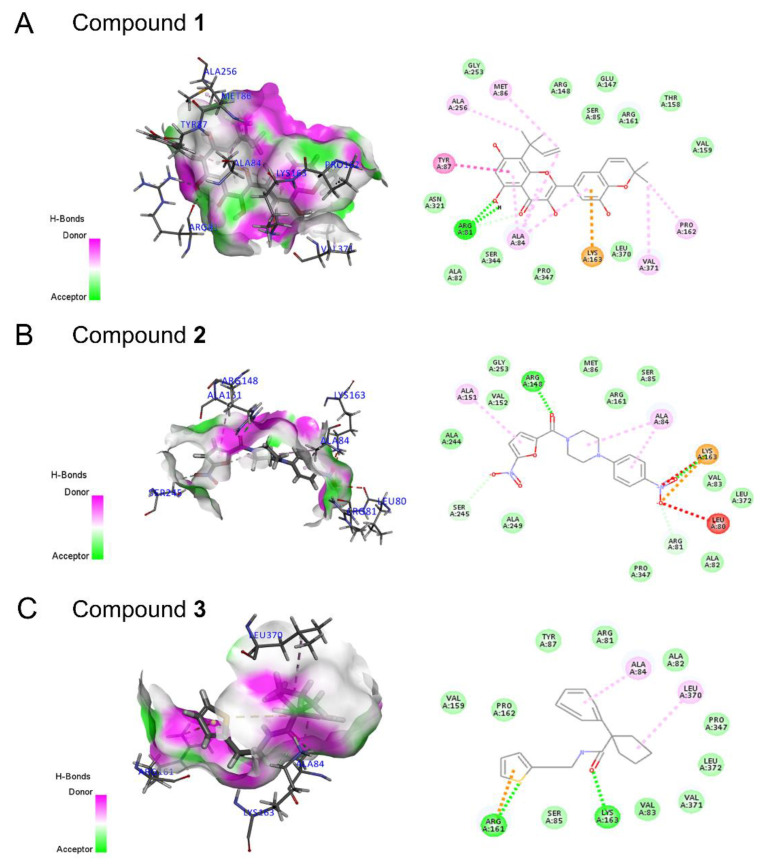
The two-dimensional interaction pattern diagrams and three-dimensional dock poses showing receptor-ligand interactions of three hits with Model 3 of urotensin receptor. (**A**) Compound **1**. (**B**) Compound **2**. (**C**) Compound **3**. For two-dimensional interaction schematic, the hydrogen bonds are shown as green dashed lines, the π–π stacking interactions are in pink and the pi–alkyl interactions are in light purple.

The receptor–ligand interaction and two-dimensional (2D) interaction pattern diagrams are shown for the three hits by Model 3 (Figure 7), together with their ADMET properties (Table S9). The CDOCKER energy scores of Compounds **1**, **2**, and **3** with the active site of the UT receptor were −12.32, −18.18, and −11.93, respectively. Generally, the lower the CDOCKER energy is, the more likely they are to interact. Of the three compounds, Compound **2** has the lowest CDOCKER energy score. Compound **1**, an isoflavone alcohol, had hydrogen bonds with Arg81 and formed hydrophobic binding of Pi-alkyl with PYR87, which increased the interaction between the molecule and protein. Compound **2** had H-bond interactions with Arg 168 and Lys163, and it also formed hydrophobic binding Pi-alkyl with Ala151 and Ala84. Similarly, Compound **3** had H-bond interactions with Arg 161 and Lys163 and formed hydrophobic binding Pi-alkyl with Leu370 and Ala84.

In addition, AutoDock Vina Software was also used for molecular docking among Model 3, U-II, URP, and three hits, and the results were visualized by PyMOL (Figure S2). The individual values of binding affinity with the studied ligands were −9.2, −6.2, and −7.7 kcal/mol, respectively. Among them, we found that the binding affinity of Compound **1** (−9.2 kcal/mol) was lower than that of U-II (−8.8 kcal/mol) and URP (−8.5 kcal/mol).

### 2.6. In Vitro Validation of UT Receptor Agonism

**Figure 8:**
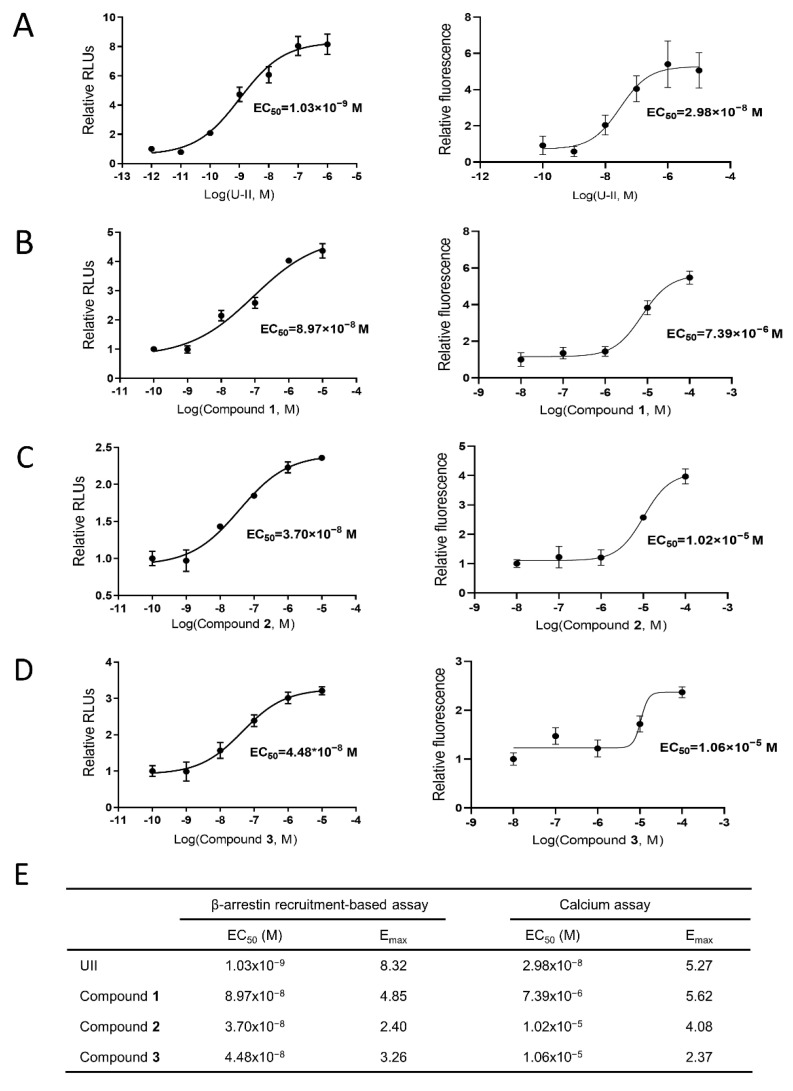
The in vitro luciferase β-arrestin recruitment-based assay and calcium assay of U-II and hits. (**A**) U-II. (**B**) Compound **1**. (**C**) Compound **2**. (**D**) Compound **3**. Left: β-arrestin recruitment-based assay; right: calcium assay. (**E**) The table recapitulated the EC_50_ and E_max_ of U-II and hits. Data represent means ± SEM, N = 4–6. Statistical analysis tested by one-way ANOVA with Dunnett’s multiple comparisons test. U-II: urotensin-II.

**Figure 9:**
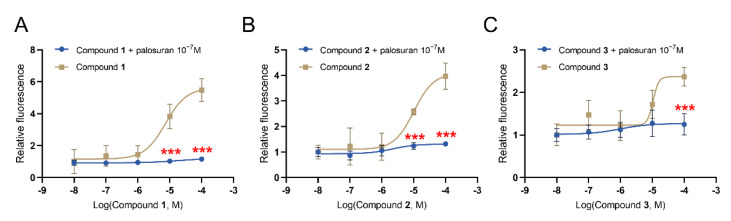
The in vitro calcium assay of hits after incubation with urotensin receptor antagonist palosuran at the concentration of 10^−7^ M. (**A**) Compound **1**. (**B**) Compound **2**. (**C**) Compound **3**. Data represent means ± SEM, N = 4–6. Statistical analysis tested by one-way ANOVA with Dunnett’s multiple comparisons test. *** *p* < 0.001.

It has been found that the activation of the UT receptor could conduct signal transduction through a G-protein-dependent pathway or a non-G-protein-dependent pathway [30]. We evaluated the activities of U-II and these three lead compounds in vitro using a luciferase β-arrestin recruitment-based assay and calcium assay. The β-arrestin recruitment results showed that the EC_50_ values of U-II, Compound **1**, Compound **2**, and Compound **3** were 1.03 × 10^−9^ M, 8.97 × 10^−8^ M, 3.70 × 10^−8^ M, and 4.48 × 10^−8^ M, respectively (Figure 8). Then, intracellular calcium mobilization via a fluorescence-based assay was used to detect UT activation. The results showed that the EC_50_ values of U-II, Compound **1**, Compound **2**, and Compound **3** were 3.52 × 10^−8^ M, 7.39 × 10^−6^ M, 1.02 × 10^−5^ M, and 1.06 × 10^−5^ M, respectively (Figure 8). As shown in Figure 8E, a table recapitulated the EC_50_ and E_max_ of U-II and hits. In parallel, preincubation with palosuran (the known UT receptor antagonist) showed that palosuran specifically reduced the increase in intracellular calcium mobilization caused by the activation of the UT receptor by the compounds (Figure 9).

### 2.7. Effect of Leads on Glucose Consumption and Lactic Acid Release

**Figure 10:**
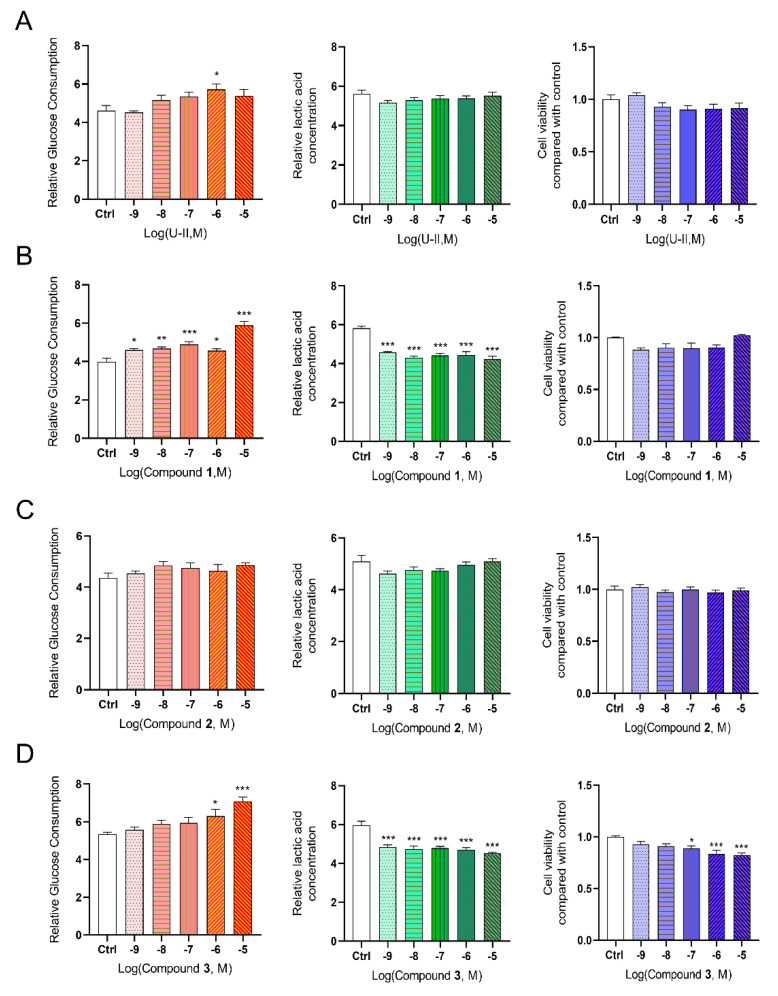
Effects of U-II and the hits on glucose consumption, lactic acid release, and 24 h cell viability in hepatic HepG2 cells in vitro. (**A**) U-II. (**B**) Compound **1**. (**C**) Compound **2**. (**D**) Compound **3**. Data represent means ± SEM, N = 6. Statistical analysis tested by one-way ANOVA with Dunnett’s multiple comparisons test. * *p* < 0.05, ** *p* < 0.01, *** *p* < 0.001. U-II: urotensin-II.

Our and others’ previous studies showed that activating of the UT receptor could affect glucose uptake and utilization [8,31,32]. In this study, we compared the effects of these three lead compounds on glucose consumption (Figure 10). The results showed that Compound **1** could significantly accelerate 48 h total glucose consumption in HepG2 cells in a concentration-dependent manner (Figure 10B). Moreover, Compound **3** significantly increased glucose consumption at higher concentrations (10^−6^ M, 10^−5^ M). Furthermore, the effects of three compounds on lactic acid release were also detected, and Compounds **1** and **3** both reduced lactic acid production. We also compared the effects of the three compounds on cell viability in vitro. Compound **1** did not affect HepG2 cell viability at the indicated concentration, and Compound **3** significantly reduced cell viability compared with the control (Figure 10B,D). However, Compound **2** showed no significant differences in glucose consumption, cell viability, or lactic acid release (Figure 10C).

## 3. Discussion

In this study, multiple approaches were combined for UT agonist discovery, including unbiased benchmark dataset construction, homologous protein modeling, virtual screening by homologous protein docking-based modeling, and pharmacophore-based modeling. Further confirmation by in vitro cellular UT receptor activation models resulted in three lead compounds.

In this study, we compared and adopted the three different modeling methods of single-based, multi-based, and threading modeling to construct homologous models for DBVS of the UT receptor. It has been previously reported that the model of the UT receptor was constructed using a molecular homology method based on the rhodopsin X-ray structure [25,33]. However, the model used was a single-template model and the identity of the protein sequence between rhodopsin and the UT receptor was low [25], which is consistent with our findings that single-template modeling was inferior among the three modeling methods. The advantage of multi-template modeling is to find the dominant structure with the target sequence among multiple sequence fragments. Based on homology, this modeling approach makes it easier to determine the structural features of conserved regions. However, it does not perform as well as threading modeling, which can correspond to similar protein structures even for dissimilar amino acid sequences and therefore does not require high template similarity and identity [34]. In this study, we found the scores of the threading model (Model 3) were superior to the others, which suggests that the threading model may be more accurate for the UT receptor.

We also constructed pharmacophore models for pharmacophore-based VS of the UT receptor. Pharmacophore-based VS is based on the assumption that a molecule with a similar structure (in terms of structure, pharmacophoric features, molecular fields, etc.) also exhibits similar behavior [35,36]. Compared with a previous report that the pharmacophores were derived from U-II [24], in this study, we used 18 structurally diverse molecules for pharmacophore generation, which may have more diverse pharmacophores, thereby increasing the sensitivity.

We also used another independent docking method (AutoDock Vina) to dock the screened compounds. Unlike the LibDock and CDocker modules in DS, AutoDock Vina is applicable not only to the docking of small molecules but also to large molecules, such as peptides U-II and URP. We found that the affinity of each compound docked to the UT receptor was close to that of U-II and URP, which helped to make the previous docking results more convincing. Moreover, from the 2D interaction pattern diagrams between the homologous models of the UT receptor and three screened compounds, we found that strong hydrogen bond interactions with amino acid residues Arg81, Arg168, Arg161, and Lys163 played key roles in the binding affinity of potential compounds with the UT receptor. Therefore, compounds with donor or acceptor groups that can form H-bonds with these residues are likely to have a better affinity. Subsequently, we evaluated the activation of GPCR downstream signaling pathways (β-arrestin pathway and G-protein-dependent pathway) with three compounds. Similar to previous reports [12,30,37], these three compounds we identified showed less agonistic potency on the β-arrestin recruitment than U-II. As for calcium assay, compound 1 behaved as UT receptor full potent agonist while compound 3 showed weak activity, which we suspected might be related to the signaling transduction bias of the UT receptor [30,38] and worthy of further study. However, as a peptide, U-II is extremely unstable and prone to inactivation, which poses a challenge for related drug development. The small molecule agonists we found are more stable and promising.

The UT receptor has attracted increasing attention in glucose and lipid metabolism [31,39,40,41]. Our previous research found that U-II increases glucose consumption in vitro and chronic administration of U-II improves whole-body glucose tolerance in high-fat-diet-fed mice [8]. Consistent with our previous observation [8], two of the lead compounds promoted glucose consumption with reduced anaerobic respiration.

## 4. Materials and Methods

### 4.1. UT Receptor Docking-Based Virtual Screening

#### 4.1.1. Homology Modeling

The amino acid sequence of the human UT receptor (NP_061822) was retrieved from the NCBI [42] website (https://www.ncbi.nlm.nih.gov/, accessed on 6 December 2022) and employed for a homology model template search using the NCBI-BLAST [42] and GPCRdb database (https://gpcrdb.org, accessed on 6 December 2022) [43]. Discovery Studio (DS) 2018 was used to generate UT receptor homology models through a single template modeling strategy. Proteins (4RWA, 4N6H, 4RWD, 5DHH, 5NDD, 4DJH, 2RH1, 6G79, and 5DHG) were selected to construct single-template UT receptor models, 10 models were generated for each template, and the highest scoring one was selected as the model for that template.

Multiple templates for the UT receptor were obtained from GPCR-Sequence-Structure-Feature-Extractor (GPCR-SSFE, http://www.ssfa-7tmr.de/ssfe2/, accessed on 6 December 2022) [44], and the database provides template suggestions and homology models of class A GPCRs.

We also used the Iterative Threading ASSEmbly Refinement web server (I-TASSER, http://zhanglab.ccmb.med.umich.edu/I-TASSER, accessed on 6 December 2022) [34,45] for the threading modeling of the UT receptor. I-TASSER is ranked as one of the top servers for protein structure prediction [46].

#### 4.1.2. Homology Model Evaluation

The stereochemical quality of the models was evaluated using the web services provided by the Molecular Biology Institute and the DOE-MBI Institute at the University of California, Los Angeles (https://saves.mbi.ucla.edu/, accessed on 6 December 2022) [28,29]. The constructed models were docked with the active compounds. The model that identified the most active compounds was used as the optimal model for subsequent virtual screening.

#### 4.1.3. UT Receptor Docking-Based Virtual Screening

The LibDock Protocol in DS 2018 was used for UT receptor DBVS [17]. According to the structural features of the receptor, the algorithm matched the pose of the ligands and then scored the binding mode of the receptor–ligand to find the most reasonable combination. In the Fast LibDock process, the ‘max hits to save’ was set as one, and the other parameters were defaulted. To filter out compounds with poor docking scores, the top-ranked molecules with scores greater than 140 points were selected for following Best LibDock. Then, the ‘conformation method’ was set to best, and the ‘minimization algorithm’ was set to the smart minimizer. Compounds with LibDock scores greater than 100 points were applied for the third-round docking using the CDOCKER protocol.

AutoDock Vina [47] was also used to evaluate the binding between the three compounds obtained from screening and the UT receptor. AutoDock Tools utilities were used to prepare the input files for AutoDock Vina, in which all the rotatable bonds of ligands were allowed to rotate freely. For docking studies, the grid box wraps the receptor protein with high resolution in the case of an unknown active site, allowing the program to search for places of probable interactions between the ligands and the receptor. Other configurations were default. The docking results were visualized with PyMOL.

### 4.2. Pharmacophore-Based Virtual Screening

#### 4.2.1. Pharmacophore Model Generation and Validation

The pharmacophore module in DS 2018 was employed to build the HipHop pharmacophore model with qualitative common features. Before building the HipHop pharmacophore model, the common features from the pharmacophore generation module “Feature Mapping” were used to identify the important chemical features of the training set compounds, such as hydrogen bond acceptors, hydrogen bond donors, hydrophobic features, and aromatic rings [22]. Conformation generation was set as best and the energy threshold was set to 10, while all the other parameters were default. A test set including active compounds and inactive compounds was used to validate whether it was able to distinguish the active compounds.

#### 4.2.2. Pharmacophore-Based Virtual Screening

DS 2018 was used to optimize compounds, and a total of 35,285 compounds were used to construct a new virtual screening 3D database. The number of conformations was set to 300, while the conformation method was set to BEST, which provided a complete and improved coverage of the conformational space by performing rigorous energy minimization and optimizing the conformations in both torsional and Cartesian space with the poling algorithm [19]. The minimum interfeature distance was set to two, and the maximum number of pharmacophores was set to 10. The remaining parameters were default values [19].

### 4.3. Benchmarking Dataset Construction

#### 4.3.1. Ligand Preparation

The UT receptor ligand data were mainly retrieved from the ChEMBL database (https://www.ebi.ac.uk/chembl/, accessed on 6 December 2022) and recorded according to activity, standard type, standard activity value, ligand efficiency (LE), canonical SMILES, etc. The criteria applied to the collection of ideal ligands were as follows: (1) to obtain ligands annotated with quantitative activity data of EC_50_ or IC_50_ ≤ 1 μM. If multiple activities were available for a single ligand, the recent data were preferred over the old. (2) To preferably obtain ligands annotated with LE data. LE is a widely used design parameter in drug discovery, which is calculated by scaling affinity by molecular size and has a nontrivial dependency on the concentration unit [48]. In general, the LE threshold was set to 0.3 kcal/mol. If the number of collected ligands was limited after applying the criterion, other ligands with incomplete or poor LE data were considered.

#### 4.3.2. Decoy Generation

A total of 196 diverse, known ligands were used to generate decoys from GPCR Ligand Library/GPCR Decoy Database using MUBD-Decoy Maker 2.0 [49,50], which is a tool to build the benchmarking sets for virtual screening on GPCRs [49]. A combination of known inactive compounds and compounds from MUBD-Decoy Maker 2.0 was used as a decoy.

### 4.4. Cell Line Culture

HTLA cells (a HEK293 cell line stably expressing a tTA-dependent luciferase reporter and a β-arrestin2-TEV fusion gene) were a gift from the laboratory of R. Axel (he has given us permission to distribute them) [51] and are maintained in DMEM supplemented with 10% FBS, 100 U/mL penicillin and 100 μg/mL streptomycin, 2 μg/mL puromycin and 100 μg/mL hygromycin B. HepG2 cells (C5 passage, human hepatoma cell line) were purchased from the National Platform of Experimental Cell Resources for Sci-Tech, Chinese Academy of Medical Sciences (Beijing, China), and 293A8 cells were the cell line HEK293A stably transfected with the UT receptor in our laboratory [52]. HepG2 and 293A8 cells were grown in high glucose (25 mmol/L) Dulbecco’s modified Eagle’s medium (DMEM, Gibco, CA, USA) supplemented with 10% fetal bovine serum (FBS, Gibco, CA, USA), 100 U/mL penicillin and 100 µg/mL streptomycin solution (Solarbio, Beijing, China). Cells were cultured in a standard humidified incubator at 37 °C under a 5% CO_2_ atmosphere. The media were changed every 2 days. Subcultures were performed at a ratio of 1:3 when cells were approximately 75–85% confluent.

### 4.5. UT Receptor Activation Experimental Assay

The β-arrestin recruitment-based experiments were performed according to previous reports with slight modifications [51]. In brief, the UT receptor plasmid was transfected into HTLA cells with the transfection reagent PEI when the cell confluence reached approximately 80%. Transfected cells were seeded in 96-well plates at a density of 1.5 × 10^5^ cells/well. After 24 h, the cells were treated with compounds diluted to the specified concentration using 10% FBS DMEM. Following the next 24 h incubation with compounds, the medium was removed and Bright-GloTM (Promega, Madison, WI, USA) reagent was added immediately. Then, the activity of firefly luciferase luminescence (RLUF) was measured using a SpectraMax M5 microplate reader (Molecular devices, LLC., San Jose, CA, USA).

Measurement of intracellular calcium mobilization was performed using Fluo-8 Calcium Assay Kit (Applygen, Beijing, China) following the manufacturer’s instructions.

### 4.6. In Vitro Biology Assay

#### 4.6.1. Cell Viability Detection

Cell viability was detected by Cell Counting Kit-8 (CCK-8) per the instructions of the technical manual (Dojindo, Kumamoto, Japan). In brief, the cells were seeded in a 96-well plate and incubated overnight in the cell culture incubator. After the cells were treated with compounds for a certain period, the CCK-8 test solution was added at 1/10 of the total volume. Then, the plates were incubated in the incubator for 2 h, and the absorbance at 450 nm was measured using a SpectraMax M5 microplate reader (Molecular Devices, LLC, San Jose, CA, USA).

#### 4.6.2. Measurements of Glucose Consumption and Lactic Acid Production

Glucose consumption detection was performed as described previously [53]. In brief, HepG2 cells in 96-well plates at a density of 8000 cells/well were treated with compounds at 37 °C, and 5% CO_2_. After the indicated duration, the media from cultured cells were taken for glucose and lactic acid detection. The glucose levels were detected by a blood-glucose kit from Biosino Biotechnology (Beijing, China). The amount of glucose consumption was calculated by subtracting the initial glucose level from the remaining glucose in the wells. The lactate content was measured using the lactic acid assay kit (Nanjing Jiancheng Bioengineering Institute, Nanjing, China) according to the manufacturer’s instructions.

### 4.7. Statistical Analysis

Data are presented as the means ± SEMs. Statistical analysis was carried out using GraphPad Prism 8.0 (GraphPad Software, Inc., San Diego, CA, USA). For statistical comparisons, an unpaired two-tailed Student’s *t* test was used as needed. The significance level was set at *p* ≤ 0.05.

## 5. Conclusions

In conclusion, in this study, we used an approach combining an unbiased benchmark dataset with DBVS and pharmacophore-based VS, together with in vitro biological evaluation, to discover novel agonists targeting on the UT receptor. According to the docking, pharmacophore, and cellular-based activity analysis, we found three compounds with UT receptor agonist activities. Compound 1 could also promote glucose utilization in HepG2 hepatocytes without obvious cytotoxic effects. Our study suggests that the approach we adopted would greatly improve the screening efficiency for drug discovery for the UT receptor.

## Data Availability

The datasets used during the current study are available from the corresponding author on reasonable request.

## Supplementary Materials

The following are available online at https://www.mdpi.com/article/10.3390/molecules27248692/s1, Figure S1: Each particular helix for multi-template based homologous model of urotensin receptor; Figure S2: Docking results between U-II, URP, three hits, and Model 3 of urotensin receptor using AutoDock Vina. (A) U-II, (B) URP, (C) Compound **1**, (D) Compound **2**, (E) Compound **3**; Table S1: 196 ligands targeting the urotensin receptor collected for the construction of the benchmarking dataset; Table S2: Identity of 10 templates for single-template modeling and RMSD value of 10 homology models; Table S3: Template suggestions and similarity for urotensin receptor multi-template modeling; Table S4: Evaluation results of models constructed based on three different modeling methods by Ramachandran plot, overall quality factor, and verify 3D. STM: single-template model. MTM: multi-template model. TDM: threading model.; Table S5: Docking results of 11 generated models with active ligands using the LibDock module of Discovery Studio. STM: single-template model. MTM: multi-template model. TDM: threading model.; Table S6: RMSD comparison between different modeling models; Table S7: ChEMBL ID, two-dimensional structure, and EC_50_ of 14 training set molecules; Table S8: The parameters of 10 common features of pharmacophore; Table S9: The ADMET properties of three screened chemical compounds.

## Sample Availability

Samples of the compounds **1**, **2**, and **3** are available from the authors.
