# Supplementary material for: Combination of Docking-Based and Pharmacophore-Based Virtual Screening Identifies Novel Agonists That Target the Urotensin Receptor
Source: Molecules. 2022 Dec 8;27(24):8692. doi: 10.3390/molecules27248692 (PMC9788431; doi:10.3390/molecules27248692)
Supplement: Supplementary file 1 [file molecules-27-08692-s001.zip › Table S1-S9.pdf]

Table S1. 196 ligands targeting UT receptor collected for the construction of the benchmarking dataset.

No.	ChEMBL ID	Activity	Activity Parameter	Activity Value(nM)
1	CHEMBL437430	agonist	EC_{50}	1.09
2	CHEMBL2372899	agonist	EC_{50}	1.80
3	CHEMBL53181	agonist	EC_{50}	1.80
4	CHEMBL385616	agonist	EC_{50}	1.80
5	CHEMBL2372897	agonist	EC_{50}	1.90
6	CHEMBL298803	agonist	EC_{50}	2.30
7	CHEMBL427632	agonist	EC_{50}	2.40
8	CHEMBL408106	agonist	EC_{50}	2.50
9	CHEMBL2372896	agonist	EC_{50}	2.70
10	CHEMBL414256	agonist	EC_{50}	3.00
11	CHEMBL2372907	agonist	EC_{50}	3.20
12	CHEMBL2372902	agonist	EC_{50}	3.30
13	CHEMBL1627325	agonist	EC_{50}	3.47
14	CHEMBL216349	agonist	EC_{50}	3.60
15	CHEMBL385962	agonist	EC_{50}	3.80
16	CHEMBL4100932	agonist	EC_{50}	4.17
17	CHEMBL4066696	agonist	EC_{50}	5.01
18	CHEMBL2372901	agonist	EC_{50}	5.70
19	CHEMBL192359	agonist	EC_{50}	5.95
20	CHEMBL4076150	agonist	EC_{50}	6.17
21	CHEMBL607809	agonist	EC_{50}	6.94
22	CHEMBL4071437	agonist	EC_{50}	7.08
23	CHEMBL593612	agonist	EC_{50}	8.51
24	CHEMBL4071437	agonist	EC_{50}	8.51
25	CHEMBL593663	agonist	EC_{50}	10.07
26	CHEMBL4076150	agonist	EC_{50}	10.72
27	CHEMBL4066696	agonist	EC_{50}	10.96

28	CHEMBL607810	agonist	EC_{50}	12.58
29	CHEMBL607811	agonist	EC_{50}	13.20
30	CHEMBL4100932	agonist	EC_{50}	14.45
31	CHEMBL4208184	agonist	EC_{50}	15.14
32	CHEMBL608099	agonist	EC_{50}	16.72
33	CHEMBL608098	agonist	EC_{50}	20.55
34	CHEMBL573392	agonist	EC_{50}	22.91
35	CHEMBL207823	agonist	EC_{50}	32.36
36	CHEMBL573857	agonist	EC_{50}	32.36
37	CHEMBL572439	agonist	EC_{50}	43.65
38	CHEMBL1171808	agonist	EC_{50}	51.29
39	CHEMBL412753	agonist	EC_{50}	53.00
40	CHEMBL203626	agonist	EC_{50}	66.07
41	CHEMBL573569	agonist	EC_{50}	74.13
42	CHEMBL608097	agonist	EC_{50}	75.66
43	CHEMBL572882	agonist	EC_{50}	75.86
44	CHEMBL583981	agonist	EC_{50}	77.62
45	CHEMBL204988	agonist	EC_{50}	77.62
46	CHEMBL204989	agonist	EC_{50}	77.62
47	CHEMBL578654	agonist	EC_{50}	104.71
48	CHEMBL383698	agonist	EC_{50}	109.65
49	CHEMBL4100932	agonist	EC_{50}	117.49
50	CHEMBL425592	agonist	EC_{50}	117.49
51	CHEMBL574527	agonist	EC_{50}	120.23
52	CHEMBL4208184	agonist	EC_{50}	120.23
53	CHEMBL574527	agonist	EC_{50}	120.23
54	CHEMBL572883	agonist	EC_{50}	125.89
55	CHEMBL228470	agonist	EC_{50}	128.82
56	CHEMBL380346	agonist	EC_{50}	134.9

57	CHEMBL229713	agonist	EC_{50}	134.9
58	CHEMBL206811	agonist	EC_{50}	144.54
59	CHEMBL1173399	agonist	EC_{50}	147.91
60	CHEMBL383326	agonist	EC_{50}	190.55
61	CHEMBL4205274	agonist	EC_{50}	190.55
62	CHEMBL2372903	agonist	EC_{50}	193.00
63	CHEMBL572398	agonist	EC_{50}	199.53
64	CHEMBL2372895	agonist	EC_{50}	233.00
65	CHEMBL192359	agonist	EC_{50}	250.00
66	CHEMBL203051	agonist	EC_{50}	251.19
67	CHEMBL535141	agonist	EC_{50}	251.19
68	CHEMBL1172223	agonist	EC_{50}	263.03
69	CHEMBL205681	agonist	EC_{50}	275.42
70	CHEMBL206551	agonist	EC_{50}	288.4
71	CHEMBL192359	agonist	EC_{50}	300.00
72	CHEMBL572391	agonist	EC_{50}	309.03
73	CHEMBL576746	agonist	EC_{50}	316.23
74	CHEMBL535141	agonist	EC_{50}	316.23
75	CHEMBL575629	agonist	EC_{50}	346.74
76	CHEMBL573575	agonist	EC_{50}	363.08
77	CHEMBL229710	agonist	EC_{50}	371.54
78	CHEMBL229714	agonist	EC_{50}	380.19
79	CHEMBL228471	agonist	EC_{50}	398.11
80	CHEMBL207783	agonist	EC_{50}	407.38
81	CHEMBL374817	agonist	EC_{50}	426.58
82	CHEMBL582835	agonist	EC_{50}	436.52
83	CHEMBL229923	agonist	EC_{50}	436.52
84	CHEMBL229764	agonist	EC_{50}	457.09
85	CHEMBL573640	agonist	EC_{50}	457.09

86	CHEMBL206832	agonist	EC_{50}	467.74
87	CHEMBL1173172	agonist	EC_{50}	501.19
88	CHEMBL387661	agonist	EC_{50}	512.86
89	CHEMBL205991	agonist	EC_{50}	524.81
90	CHEMBL203937	agonist	EC_{50}	524.81
91	CHEMBL578853	agonist	EC_{50}	524.81
92	CHEMBL575819	agonist	EC_{50}	537.03
93	CHEMBL390792	agonist	EC_{50}	575.44
94	CHEMBL387694	agonist	EC_{50}	588.84
95	CHEMBL229709	agonist	EC_{50}	602.56
96	CHEMBL573574	agonist	EC_{50}	645.65
97	CHEMBL574731	agonist	EC_{50}	645.65
98	CHEMBL572467	agonist	EC_{50}	660.69
99	CHEMBL1172763	agonist	EC_{50}	676.08
100	CHEMBL1173492	agonist	EC_{50}	691.83
101	CHEMBL1173081	agonist	EC_{50}	724.44
102	CHEMBL572454	agonist	EC_{50}	741.31
103	CHEMBL575630	agonist	EC_{50}	812.83
104	CHEMBL572392	agonist	EC_{50}	812.83
105	CHEMBL3104642	antagonist	IC_{50}	0.000001
106	CHEMBL3104468	antagonist	IC_{50}	0.00001
107	CHEMBL3104466	antagonist	IC_{50}	0.0001
108	CHEMBL1164032	antagonist	IC_{50}	3.60
109	CHEMBL495075	antagonist	IC_{50}	4.00
110	CHEMBL567303	antagonist	IC_{50}	5.00
111	CHEMBL522770	antagonist	IC_{50}	6.00
112	CHEMBL567713	antagonist	IC_{50}	8.00
113	CHEMBL522770	antagonist	IC_{50}	8.00
114	CHEMBL3104644	antagonist	IC_{50}	9.00

115	CHEMBL3104464	antagonist	IC_{50}	10.00
116	CHEMBL3104471	antagonist	IC_{50}	12.00
117	CHEMBL3104637	antagonist	IC_{50}	16.98
118	CHEMBL3358682	antagonist	IC_{50}	20.00
119	CHEMBL3358685	antagonist	IC_{50}	20.00
120	CHEMBL3104465	antagonist	IC_{50}	21.00
121	CHEMBL3977330	antagonist	IC_{50}	25.00
122	CHEMBL3358679	antagonist	IC_{50}	26.00
123	CHEMBL3104463	antagonist	IC_{50}	27.54
124	CHEMBL3104462	antagonist	IC_{50}	29.51
125	CHEMBL2348502	antagonist	IC_{50}	34.00
126	CHEMBL3358686	antagonist	IC_{50}	42.00
127	CHEMBL2348501	antagonist	IC_{50}	43.00
128	CHEMBL3104638	antagonist	IC_{50}	46.00
129	CHEMBL567759	antagonist	IC_{50}	47.00
130	CHEMBL578207	antagonist	IC_{50}	51.00
131	CHEMBL3896625	antagonist	IC_{50}	51.00
132	CHEMBL568637	antagonist	IC_{50}	53.00
133	CHEMBL3924660	antagonist	IC_{50}	53.00
134	CHEMBL3104639	antagonist	IC_{50}	58.00
135	CHEMBL568123	antagonist	IC_{50}	59.00
136	CHEMBL3104461	antagonist	IC_{50}	60.00
137	CHEMBL3358680	antagonist	IC_{50}	60.00
138	CHEMBL1163360	antagonist	IC_{50}	67.00
139	CHEMBL3968841	antagonist	IC_{50}	74.00
140	CHEMBL3358674	antagonist	IC_{50}	78.00
141	CHEMBL3915670	antagonist	IC_{50}	86.00
142	CHEMBL3987058	antagonist	IC_{50}	87.00
143	CHEMBL571535	antagonist	IC_{50}	88.00

144	CHEMBL3104467	antagonist	IC_{50}	88.02
145	CHEMBL3906682	antagonist	IC_{50}	100.00
146	CHEMBL2348523	antagonist	IC_{50}	100.00
147	CHEMBL3957806	antagonist	IC_{50}	100.00
148	CHEMBL2348524	antagonist	IC_{50}	120.00
149	CHEMBL3979822	antagonist	IC_{50}	130.00
150	CHEMBL567075	antagonist	IC_{50}	140.00
151	CHEMBL3358683	antagonist	IC_{50}	140.00
152	CHEMBL3898238	antagonist	IC_{50}	140.00
153	CHEMBL3907266	antagonist	IC_{50}	140.00
154	CHEMBL567736	antagonist	IC_{50}	150.00
155	CHEMBL566433	antagonist	IC_{50}	150.00
156	CHEMBL3358667	antagonist	IC_{50}	160.00
157	CHEMBL366221	antagonist	IC_{50}	170.00
158	CHEMBL2348522	antagonist	IC_{50}	180.00
159	CHEMBL3104640	antagonist	IC_{50}	181.00
160	CHEMBL3358684	antagonist	IC_{50}	190.00
161	CHEMBL2348511	antagonist	IC_{50}	191.00
162	CHEMBL567949	antagonist	IC_{50}	220.00
163	CHEMBL568165	antagonist	IC_{50}	240.00
164	CHEMBL2348504	antagonist	IC_{50}	245.00
165	CHEMBL565580	antagonist	IC_{50}	260.00
166	CHEMBL3104643	antagonist	IC_{50}	277.00
167	CHEMBL3949719	antagonist	IC_{50}	310.00
168	CHEMBL567303	antagonist	IC_{50}	320.00
169	CHEMBL2348528	antagonist	IC_{50}	350.00
170	CHEMBL3104459	antagonist	IC_{50}	361.00
171	CHEMBL3104459	antagonist	IC_{50}	363.08
172	CHEMBL565416	antagonist	IC_{50}	410.00

173	CHEMBL2348505	antagonist	IC_{50}	440.00
174	CHEMBL2348519	antagonist	IC_{50}	450.00
175	CHEMBL3358671	antagonist	IC_{50}	450.00
176	CHEMBL566366	antagonist	IC_{50}	500.00
177	CHEMBL578203	antagonist	IC_{50}	500.00
178	CHEMBL571537	antagonist	IC_{50}	510.00
179	CHEMBL2348515	antagonist	IC_{50}	520.00
180	CHEMBL2348520	antagonist	IC_{50}	550.00
181	CHEMBL3915656	antagonist	IC_{50}	560.00
182	CHEMBL3920751	antagonist	IC_{50}	610.00
183	CHEMBL2348518	antagonist	IC_{50}	620.00
184	CHEMBL254154	antagonist	IC_{50}	630.96
185	CHEMBL2348508	antagonist	IC_{50}	669.00
186	CHEMBL2348517	antagonist	IC_{50}	691.00
187	CHEMBL2348516	antagonist	IC_{50}	714.00
188	CHEMBL2348503	antagonist	IC_{50}	742.00
189	CHEMBL568764	antagonist	IC_{50}	760.00
190	CHEMBL2348515	antagonist	IC_{50}	765.00
191	CHEMBL3942137	antagonist	IC_{50}	770.00
192	CHEMBL2348528	antagonist	IC_{50}	800.00
193	CHEMBL2348514	antagonist	IC_{50}	821.00
194	CHEMBL2348527	antagonist	IC_{50}	840.00
195	CHEMBL3358673	antagonist	IC_{50}	930.00
196	CHEMBL2348519	antagonist	IC_{50}	950.00

Table S2. Identity of ten templates for single-template modelling and RMSD value of ten homology models.

Template	Identity (%)	RMSD
4N6H	28.17	0.273
5DHG	28.98	0.179
5DHH	28.98	0.205
4EJ4	27.27	3.986
5NDD	22.92	2.730
4DJH	27.02	29.839
4RWA	28.27	0.469
4RWD	28.27	0.272
2RH1	24.07	29.078
6G79	22.11	1.922

Table S3. Template suggestions and similarity for UT receptor multi-templates modelling.

**Helix**	**Templates**	**Helix sequence similarity (%)**
TMH1	4EJ4	43.33
TMH2	4EA3	62.96
TMH3	4MBS	64.71
TMH4	5NDD	58.33
TMH5	2RH1	48.28
TMH6	4NTJ	46.67
TMH7	5UEN	53.85
H8	1U19	45.45

Table S4. Evaluation results of models constructed based on three different modelling methods by ramachandran plot, overall quality factor, and verify 3D. STM: single-template model; MTM: multi-templates model; TDM: threading model.

No.	Model	Ramachandran Plot				Overall Quality Factor	Verify 3D
		Residues in most favored regions	Residues in addition allowed regions	Residues in generally allowed regions	Residues in disallowed regions		
1	STM-1	95.6%	3.6%	5.8%	0.0%	98.1685	62.32%
2	STM-2	93.5%	4.5%	1.2%	0.8%	92.1348	60.43%
3	STM-3	95.5%	4.0%	0.4%	0.4%	97.3684	60.5%
4	STM-4	95.1%	4.5%	0.4%	0.0%	96.0784	51.44%
5	STM-5	93.2%	5.6%	1.2%	0.0%	88.3636	62.54%
6	STM-6	94.8%	4.8%	0.4%	0.0%	93.7269	55.83%
7	STM-7	95.6%	4.0%	0.4%	0.0%	97.8022	71.63%
8	STM-8	93.9%	5.7%	0.0%	0.4%	95.0943	64.41%
9	STM-9	94.6%	5.1%	0.2%	0.0%	95.8673	60.41%
10	STM-10	93.5%	5.3%	1.2%	0.0%	69.8885	44.61%
11	MTM-1	93.9%	4.9%	1.2%	0.0%	95.149	63.80%
12	TDM-1	81.1%	15.5%	1.2%	2.2%	84.5144	58.87%
13	TDM -2	79.2%	13.7%	4.0%	3.1%	86.3636	60.93%
14	TDM -3	77.6%	16.5%	3.1%	2.8%	84.1689	64.52%
15	TDM -4	81.4%	14.6%	2.2%	1.9%	93.9663	62.72%
16	TDM -5	75.5%	18.6%	3.1%	2.8%	88.9764	50.90%

Table S5. Docking results of the 11 generated models with active ligands using Libdock module of Discovery studio. STM: single-template model; MTM: multi-templates model; TDM: threading model.

Model	Conformers generated	Poses docked	Ligands failed to dock
STM-1	32513	11584	0
STM-3	32513	4327	6
STM-4	32513	6983	12
STM-2	32513	8947	24
STM-5	32513	1976	50
MTM-1	32513	596	46
TDM -1	32513	714	40
TDM -2	32513	9914	42
TDM -3	32513	11541	10
TDM -4	32513	16442	0
TDM -5	32513	11427	0

Table S6. RMSD comparison between different modelling models.

RMSD	Model 1	Model 2	Model 3	Model 4
Model 1	0	2.662	1.099	3.004
Model 2	2.662	0	2.486	2.933
Model 3	1.099	2.486	0	2.808
Model 4	3.004	2.933	2.808	0

Table S7. ChEMBL ID, two-dimensional structure, and EC_{50} of 14 training set molecules.

Molecule's	Molecule ChEMBL ID	Molecular structure	Activity value EC_{50} (M)
Molecule 1	CHEMBL437430 (UII)	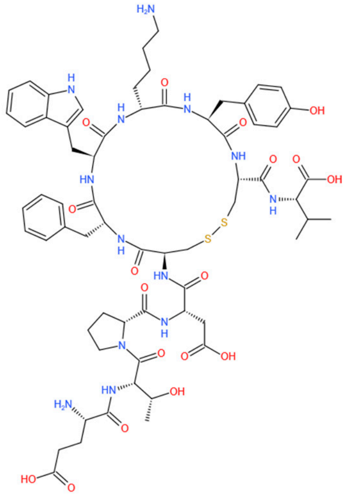	$1.09*10^{-9}$
Molecule 2	CHEMBL380346	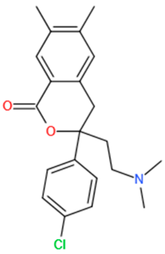	$1.35*10^{-7}$
Molecule 3	CHEMBL574304	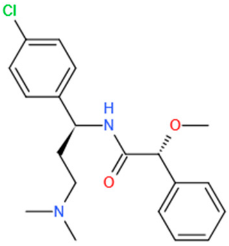	$8.71*10^{-7}$
Molecule 4	CHEMBL572467	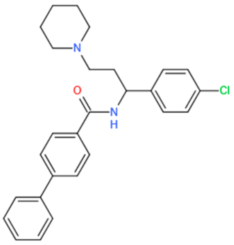	$6.61*10^{-7}$
Molecule 5	CHEMBL4071437	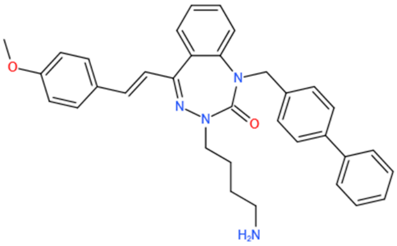	$7.08*10^{-8}$

Molecule 6	CHEMBL205991	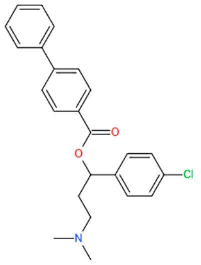	$5.25*10^{-7}$
Molecule 7	CHEMBL4205274	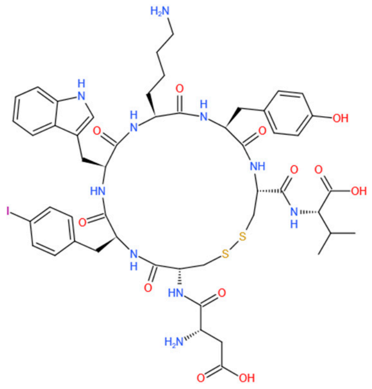	$1.91*10^{-7}$
Molecule 8	CHEMBL425592	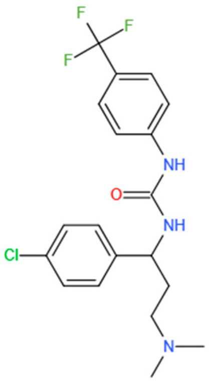	$1.17*10^{-7}$
Molecule 9	CHEMBL192359	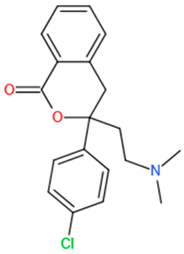	$5.95*10^{-9}$
Molecule 10	CHEMBL387694	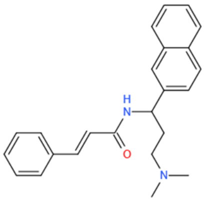	$5.89*10^{-7}$

Molecule 11	CHEMBL204989	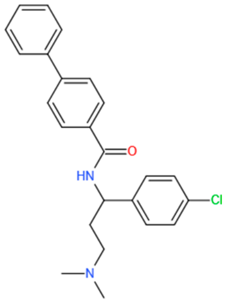	$7.76*10^{-8}$
Molecule 12	CHEMBL437110	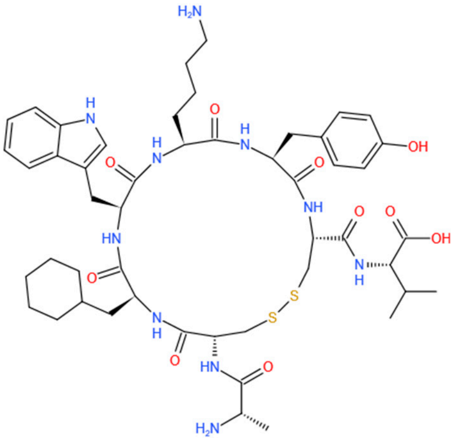	$9.33*10^{-7}$
Molecule 13	CHEMBL203051	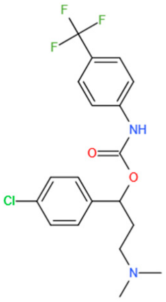	$2.51*10^{-7}$
Molecule 14	CHEMBL574527	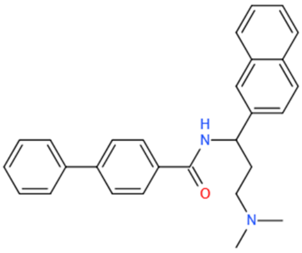	$1.20*10^{-7}$

Table S8. The parameters of ten common features of pharmacophore.

Number	Features	Score
1	HHHA	132.321
2	HHHA	125.364
3	HHHA	122.702
4	HHHA	120.363
5	HHHA	115.667
6	HHHA	115.200
7	HHHA	114.628
8	HHHA	113.562
9	HHHA	112.886
10	HHHA	110.690

Table S9. The ADMET properties of three screened chemical compounds.

Compound's	Solubility	Absorption level	BBB level	PPB(AlogP98)	CYP2D6 inhibitor	Hepatotoxic Applicability
Compound 1	-5.17	1	4	4.13	False	True
Compound 2	-3.59	0	4	2.18	False	True
Compound 3	-4.58	0	1	3.84	False	False
