# Supplementary figures and images for: Combination of Docking-Based and Pharmacophore-Based Virtual Screening Identifies Novel Agonists That Target the Urotensin Receptor
Source: Molecules. 2022 Dec 8;27(24):8692. doi: 10.3390/molecules27248692 (PMC9788431; doi:10.3390/molecules27248692)

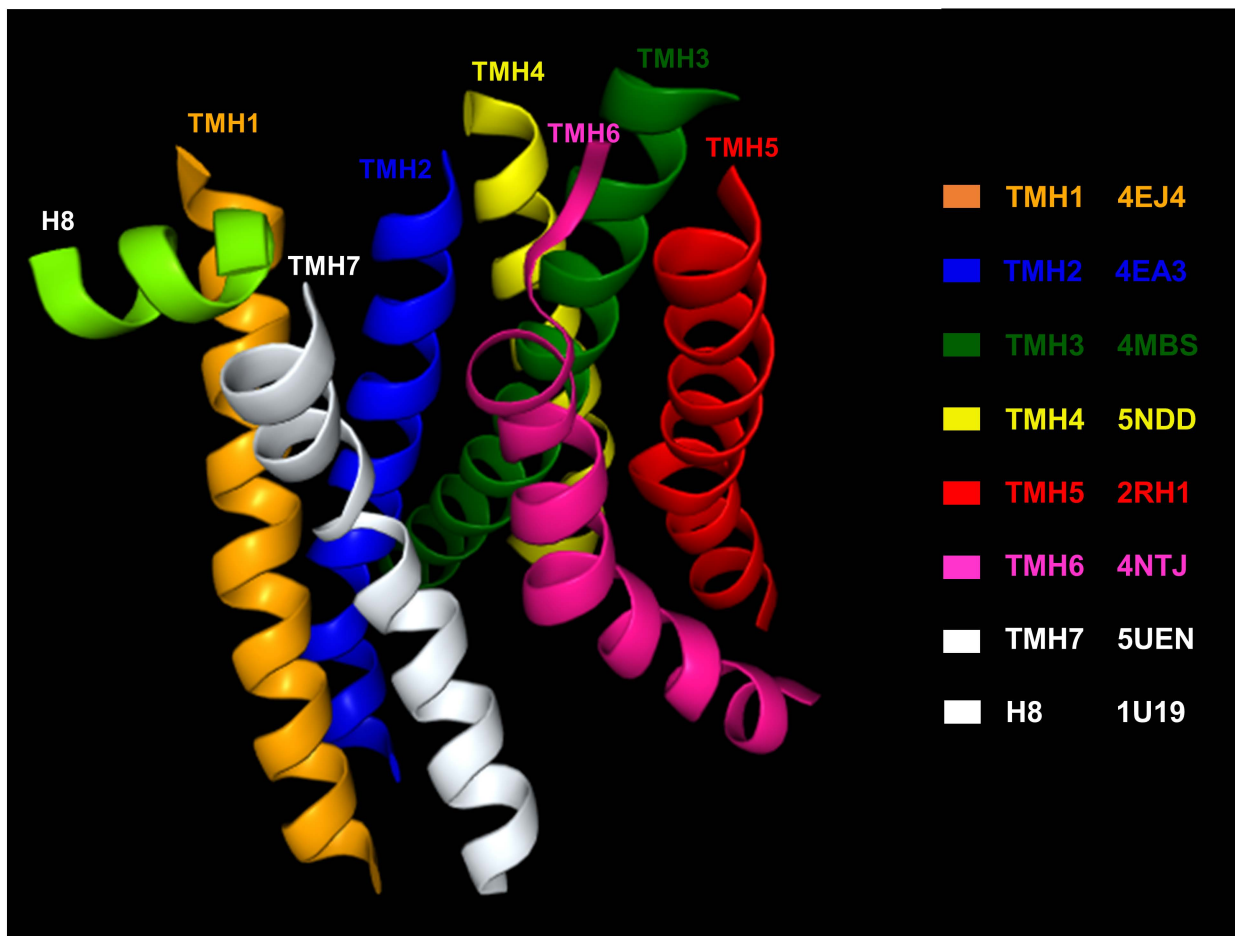

Supplement: Supplementary file 1 [file molecules-27-08692-s001.zip › Figure S1.pdf]

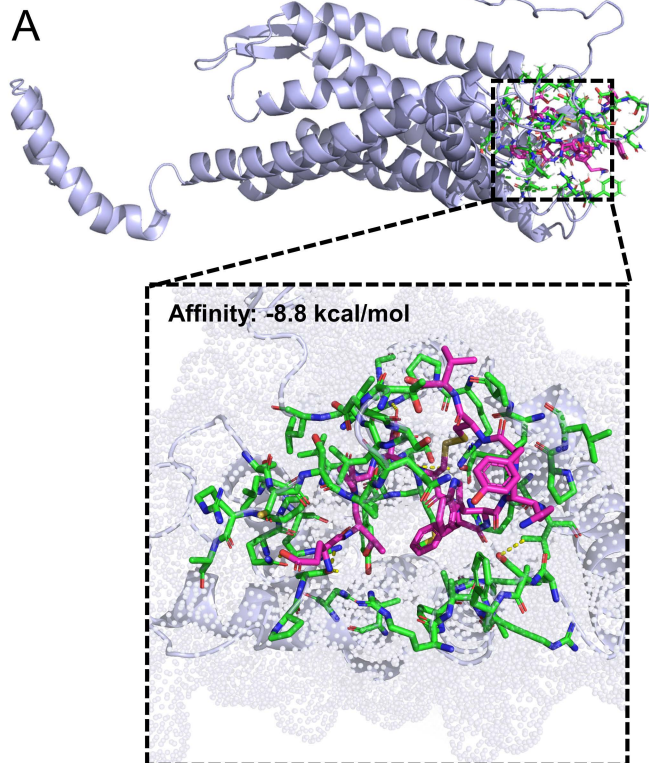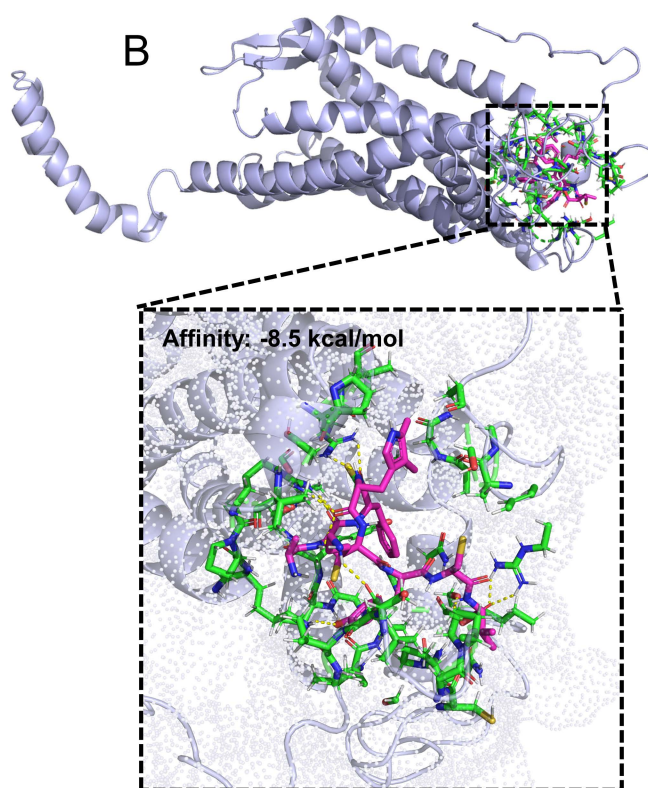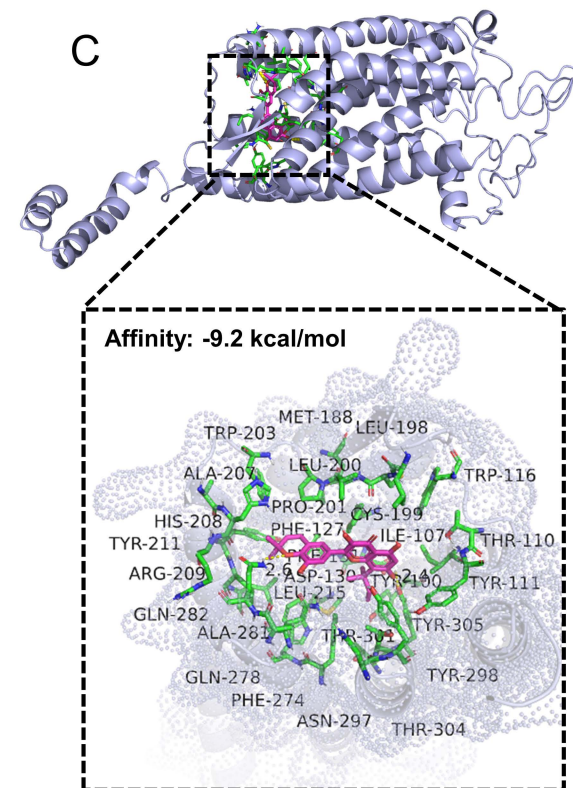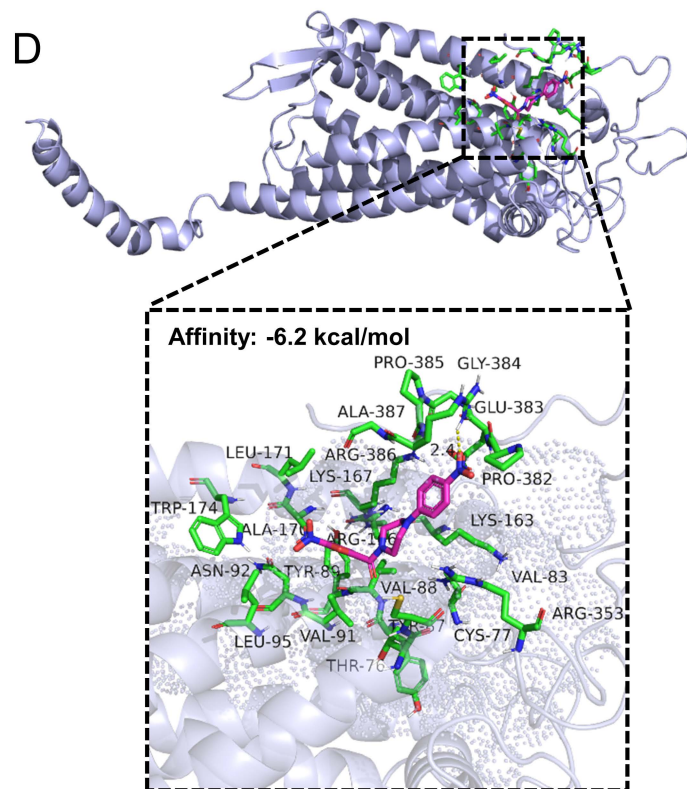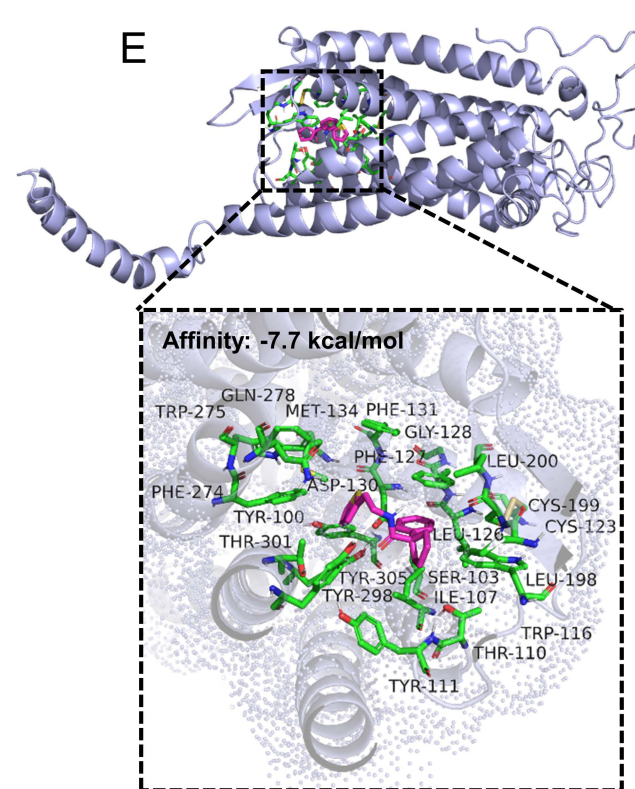

Supplement: Supplementary file 1 [file molecules-27-08692-s001.zip › Figure S2.pdf]
